# Nasal carriage of Methicillin-Resistant *Staphylococcus aureus* among sympatric free-ranging domestic pigs and wild Chlorocebus pygerythrus in a rural African setting

**DOI:** 10.1186/s12917-022-03212-9

**Published:** 2022-03-16

**Authors:** John Bosco Kalule, Valeria Zalwango Nakintu, Simon Peter SSendawula

**Affiliations:** College of Veterinary Medicine Animal Resources and Biosecurity. Department of Biotechnical and Diagnostic Sciences, Makerere University, Kampala, Uganda

**Keywords:** Antimicrobial resistance, Environment, *S. aureus*

## Abstract

**Background:**

Methicillin Resistant *Staphylococcus aureus* (MRSA) nasal carriage in domestic pigs and vervet monkeys is a risk factor for subsequent severe infections in domestic pigs and for dissemination to the human population. This study assessed nasal carriage of MRSA in domestic pigs and sympatric vervet monkeys in a rural African village during an outbreak of a virus hemorrhagic fever suspected to be contracted from wild primates.

This study was conducted during the 2012 Ebola outbreak to determine nasal carriage of MRSA in free-ranging domestic pigs and sympatric freely roaming vervet monkeys using conventional methods. *Staphylococcus aureus* (*S. aureus*) isolated from the anterior nares were tested for susceptibility to commonly used antibiotics and conventional PCR was used to confirm methicillin resistance. The MRSA strains were then genotyped using SCCmec typing.

**Results:**

Overall, there was a high level of resistance to tetracycline [90% (63/70) in pigs and 67% (10/15) in vervet monkeys], trimethoprim/sulphamethoxazole [90% (63/70) in pigs and 67% (10/15) in vervet monkeys], and penicillin [83% (58/70) in pigs and 67% (10/15) in vervet monkeys]. Most of the MRSA strains (91.6%, 11/12) were of the SCCmec type I [1B] genotype.

**Conclusion:**

The nasal carriage of drug resistant *S. aureus* in freely roaming domestic and wild animals presents a risk for widespread environmental spread of antimicrobial resistance thus presenting a risk for treatment failure in domestic animals, wild animals, and humans.

## Background

Methicillin-resistant *S. aureus* (MRSA) infections are a global concern with a considerable effect on treatment outcomes and costs [1]. The methicillin resistance gene (*mecA* gene) located in the Staphylococcal Cassette Chromosome mec (SCCmec) is responsible for the synthesis of an altered penicillin binding protein (PBP) 2a which in turn confers cross-resistance to beta-lactam antibiotics [2]. Animals play a role in the epidemiology of MRSA infection and colonization in humans [3]. The Livestock Associated MRSA (CC 398) has been documented to colonize pigs and less commonly poultry and cattle and it commonly affects veterinarians and farmers in areas with huge pig populations through direct animal contact, environmental contamination, or meat consumption [4]. Given the high extent of low host-specificity among many of the MRSA clonal lineages, it may not always be beneficial to categorize MRSA into Hospital Acquired, Community Acquired and Livestock associated [5]; especially in the developing countries where the animal- human interface is fluid. In Africa, PVL-positive MRSA (ST5) was described in nasal samples of pigs from Senegal [6, 7] and MRSA ST88 from pigs and humans in Nigeria showed high genetic similarity [8].

In Uganda, MRSA has been observed in foods of animal origin such as milk [9] and was particularly highly prevalent in domesticated pigs [10]. Interestingly, it was also observed to be highly prevalent in the hospital settings [11].

In the wild, there is minimal use of antibiotics to treat wild animals especially among wild animals such as vervet monkeys that are categorized as vermin by the Uganda Wildlife Authority (UWA); the UWA defines “vermin” as problem animals that cause damage to people, farms and other assets.

During the dry season, vervet monkeys migrate closer to homesteads in search of food from gardens due to food scarcity in the wild. In doing so, they often come close to the free ranging domestic pigs and could potentially transmit zoonotic pathogens to humans; [12] or act as sentinels for the environmental transmission of AMR [13]. Moreover, a previous study showed a high prevalence of MRSA in both colony-born and wild vervet monkeys [14]. There is no sufficient data on nasal carriage of MRSA among wild vervet monkeys and sympatric free range domestic pigs in rural African settings. This study investigated nasal carriage of MRSA among free ranging domestic pigs and sympatric wild vervet monkeys in a rural African village.

## Results

*S. aureus* was isolated from all the pigs (70/70) and free ranging vervet monkeys (15/15). Of the isolates from pigs, 11.4% (8/70) were resistant to cefoxitin and oxacillin while 26.7% (4/15) of the isolates from vervet monkeys were resistant to cefoxitin and oxacillin. Resistance to cefoxitin and oxacillin was confirmed using the *mecA* PCR. There was no significant difference in the occurrence of MRSA in the wild vervet monkeys and the free ranging domestic pigs (*p* = 0.203).

Overall, there was a high level of resistance to tetracycline [90% (63/70) in pigs, 67% (10/15) in vervet monkeys], trimethoprim/sulphamethoxazole [90% (63/70) in pigs, 67% (10/15) in vervet monkeys], and penicillin [83% (58/70) in pigs, 67% (10/15) in vervet monkeys] (Table 1).

**Table 1:** Percentage resistant for isolates from free ranging domestic pigs and wild vervet monkeys

Antibiotic (µg)	Domestic pigs (%R, *n* = 70)	Vervet Monkeys (%R, *n* = 15)
Penicillin G (10U)	83%, 58/70	67%, 10/15
Oxacillin (1 µg)	11.4%, 8/70	26.7%, 4/15
Cefoxitin (30 µg)	11.4%, 8/70	26.7%, 4/15
Erythromycin (15 µg)	1.4%, 1/70	0.00%, 0/15
Clindamycin (2 µg)	1.4%, 1/70	0.00%,0/15
Gentamicin (10 µg)	1.4%, 1/70	0.00%, 0/15
Trimethoprim/ Sulfamethoxazole (25 µg)	90%, 63/70	67%, 10/15
Tetracycline (30 µg)	90%, 63/70	67%, 10/15
Chloramphenicol (30 µg)	1.4%, 1/70	0.00%, 0/15
Ciprofloxacin (5 µg)	1.4%, 1/70	0.00%, 0/15
Fusidic acid (10 µg)	0.00%, 0/70	0.00%, 0/15
Rifampicin (30 µg)	2.9%, 2/70	6.7%, 1/15
Linezolid (30 µg)	2.9%, 2/70	0.00%, 0/15
Tigecycline (15 µg)	0.00%, 0/70	0.00%, 0/15
^a^Vancomycin	0.00%, 0/70	0.00%, 0/15

^a^susceptibility/resistance to vancomycin was based on VITEK 2

### SCCmec typing of MRSA from vervet monkeys and free-ranging domestic pigs

**Fig. 1:**
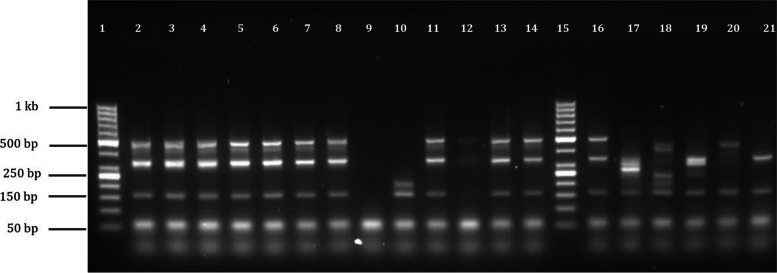
Characterization of the SCCmec types of methicillin resistant S. aureus (MRSA) control strains and the 12 MRSA strains. Lanes 2–14 contain MRSA strains from vervets and free-ranging pigs (lane 9 is a negative control). Lanes 16–21 contain MRSA control strains carrying SCCmec types I-VI; lane 16, COL (type I [1B]), lane 17, BK2464 (type II [2A]), lane 18, ANS46 (type III [3A]), lane 19, MW2 (type IV [2B]), lane 20, WIS (Type V [5C]) and lane 21, HDE288 (type VI [4B]). Lane 9 contains a non-template negative control, lanes 1 and 15 contain GeneRuler™ DNA molecular weight ladder (ThermoFisher Scientific Inc., MA, USA). Samples 2–8, and 11–14 amplified similar banding patterns as the control strain COL (162 bp, 342 bp, and 495 bp), Sample 10 (from vervet monkeys) amplified two bands (162 bp (mecA) and 209 bp (mecI))

The PCR amplification profiles for the 11 MRSA strains were similar to, but distinct from the amplification profile of the SCCmec type I [1B] control strain COL (Fig. 1). COL amplified three bands sized 495 bp, 342 bp and 162 bp, and the identified MRSA strains showed an additional band (449 bp) which corresponds to a band amplified by ccrC primers from SCCmec type V [5C] control strains (Fig. 1). The other MRSA strain was non-typeable as amplification of a 209 bp fragment which corresponds to the mecI region in SCCmec type II and III control strains was observed.

## Discussion

In rural African villages, there is a significant level of interaction between wild animals, domestic animals (especially those that are free ranging) and humans. There is no physical barrier between the wild animals and the human settlements in Uganda and there is a significant level of interaction between the vervet monkeys and free ranging domestic pigs.

During the dry season when vervet monkey food is relatively scarce in the wild, vervets migrate closer to homesteads in search of food from gardens thus posing a threat of zoonotic disease transmission to the public [12]; or risk picking up a zooanthroponosis such as tuberculosis from fellow primates – the humans [15].

High rates of resistance to clinically relevant antibiotics have been reported in the communities and it would be expected that non-human primates like the vervet monkeys living at the human-animal interface could potentially play an ecologically positive role in the reestablishment of susceptible bacteria after removal of drug pressure [16].

This study was conducted to figure out the extent of MRSA nasal carriage among sympatric vervet monkeys and free ranging domestic pigs in order to assess the level of AMR transmission risk that they pose to the nearby human communities.

Contrary to a previous study which showed a 29.7% prevalence of MRSA in domestic pigs in the South-Western region of Uganda [10], this study found a lower prevalence of 11.4%. Besides being done in a different part of Uganda, the difference in resistance rates could be explained by the difference in pig husbandry methods used – this study focused on pigs that are free-ranging. The tendency to use antibiotics is higher for intensively raised pigs as opposed to the free ranging pigs.

There was no significant difference in the prevalence of MRSA between the free-ranging domestic pigs and wild vervet monkeys; this outcome could be attributed to the small sample sizes used in this study. SCCmec typing of the confirmed MRSA strains from vervet monkeys and free-ranging domestic pigs showed that the dominant MRSA genotype at the vervet monkey -free-ranging pigs interface seems to be the SCCmec type I. Confirmation of the dominant genotype at this interface would require a larger sample size. Since there is hardly any use of antibiotics in these vervet monkey populations, the occurrence of MRSA in them is a public health concern which could be explained by the destruction of the natural ranges chiefly for agriculture and human settlement – a sequelae of a rapidly increasing human population in this region. Consequently, this has impacted the level of interaction between vervets and humans as well as with domestic animals especially those that are free ranging. Additionally, the existence of vervets in colonies of 10 to 50 might increase the likelihood of spread of different antibiotic resistance phenotypes to those in the same colony. The exact routes of spread of antibiotic resistance in this niche are not properly understood; notably, this study did not further genotype the isolates from vervets and the free ranging domestic pigs to infer similarity.

Although previous studies have shown that antibiotic resistance is commonplace in bacteria isolated from various wildlife such as monkeys, especially resistance towards old and naturally occurring antibiotics [17], the resistance phenotype reported in this study is of clinical relevance to humans, domestic animals, and wildlife. In this study, we observed a high level of resistance to key clinically relevant antibiotics such as tetracyclines, trimethoprim/sulphamethoxazole, and penicillin – a finding reminiscent of findings of several studies on *S. aureus* from human and non-human sources in Uganda. [9, 18–20] Despite the limitation of using small sample sizes, this study could hint on widespread antimicrobial resistance in this setting especially because there is no use of antibiotics in vervet monkeys in this setting. There is need to investigate and understand the contribution of environmental factors in the spread of antimicrobial resistance in this setting and the role of free ranging domestic digs and vervet monkeys. Understanding their role would aid identification of interactions that could be interrupted to reduce the spread of resistance in the environment [21].

### Recommendations

This study was conducted in the dry season only and as much as it genotyped the MRSA strains, the dominant SCCmec type and the directionality of MRSA transfer could not be confirmed because of the small sample size attributed to the difficulty in trapping vervet monkeys. A bigger study conducted over both the wet and dry seasons, would help to figure out the extent, direction, and routes of community transmission in this area. Efforts to control antimicrobial resistance in this area must factor in the nearby wildlife communities as possible reservoirs of critical resistance phenotypes.

## Conclusions

Free ranging domestic pigs and wild vervet monkeys in this area carry *S. aureus* that are resistant to clinically relevant antibiotics. Local community antimicrobial resistance (AMR) control strategies should consider the roles of wildlife in AMR spread in similar rural African settings.

### Research methods and design

**Fig. 2:**
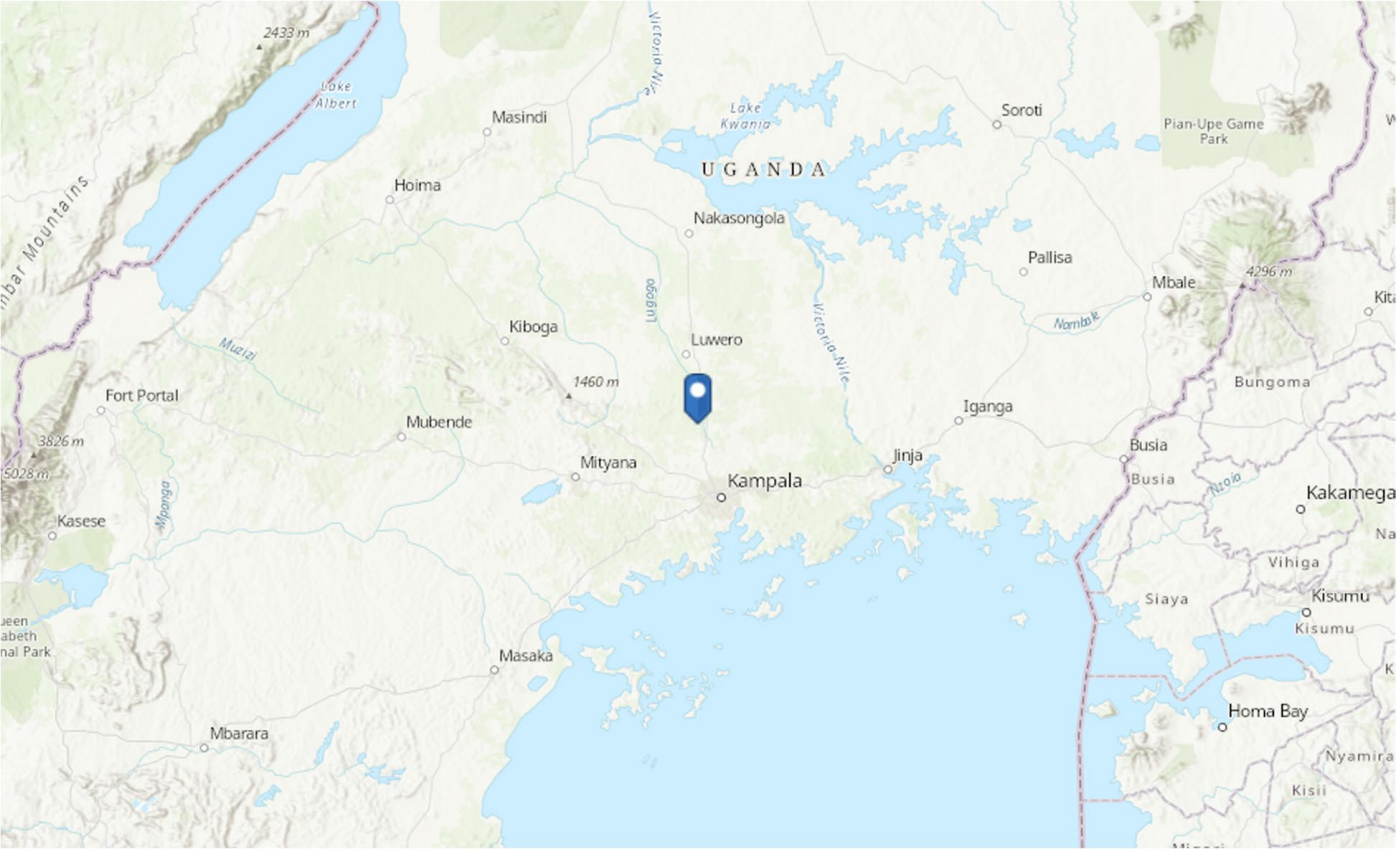
Study Area in Luwero district, Kakute village, Uganda. The blue pointer indicates the location of Kakute village in Luwero district, Uganda. The map was drawn using ArcGIS version 10.8, (https://learngis2.maps.arcgis.com)

Luwero district (Fig. 2) is in the central part of Uganda and has recently suffered two Ebola outbreaks thought to be linked to non-human primates. Kakute village is located near a local river called Lwajali which is a tributary of the Sezibwa river which drains from L. Kyoga to the North and drains downstream south to the Lake Victoria. It starts from the swamp, west of the village of Kisweera, in Mukono District, the Central Region of Uganda and It flows north to empty into River Sezibwa in Kayunga District at its border with Luweero District, east of the village of Kiziba. The region around the Lwajali river in Kakute village is lush green and habited by various forms of wildlife (and humans) including vervet monkeys. During the ecological surveillance studies to determine the non-human source of Ebola virus responsible for the 2012 Ebola outbreak, simultaneously, nasal swabs were collected to detect MRSA from free ranging pigs (Since the Reston Ebola virus was shown to affect pigs, domestic pigs were also screened as possible sources of the Ebola virus and nasal swabs were collected and tested for MRSA) and wild vervet monkeys. The domestic pigs in this region were different crossbreeds of the large white and large black, were free ranging and kept in small numbers at a subsistence level as previously described [22]. The vervets in this area were identified by ecologists from the Uganda Wildlife Authority (UWA) as Chlorocebus pygerythrus and are commonly encountered in this area destroying peoples’ gardens and are therefore categorized by UWA as ‘vermin’.

### Sample collection

Free ranging domestic pigs: We collected nasal swabs from free ranging pigs in all homesteads. The pigs were restrained using a pig strainer prior to sample collection. Ultimately, forty homesteads in Kakute village (GPS co-ordinates 0.60075, 32.5012), Luwero district were selected and visited between February 2012 and September 2013. Free-ranging pigs were restrained, and the anterior nares swabbed using sterile cotton swabs (BBL CultureSwab plus Amies Medium, Becton Dickinson, New Jersey, US). The nasal swabs were immediately transported to the veterinary microbiology laboratory at Makerere University and processed within 3 h of collection. During the same period, a team of wildlife professionals (from the Uganda Wildlife Authority) visited the nearby forests, swamps or gardens to catch wild vervet monkeys and swab their anterior nares. An air-powered darting rifle and syringe darts loaded with a mixture of Ketamine-Rompun were applied during the capture process which was done in accordance with the animal welfare regulations. The swabs were labeled with the animal species, date of collection, and sample type. The swabs were temporarily held at 11 4ºC and processed within 3 h of collection at the veterinary microbiology laboratory at Makerere University.

### Isolation of *S. aureus*

In total, 85 nasal swabs were collected from free-ranging domestic pigs (70 swabs) and wild vervet monkeys (15 swabs). The swabs were inoculated onto Mannitol Salt Agar (MSA) (Veterinary Microbiology Laboratory, College of Veterinary Medicine Animal resources and Biosecurity, Makerere University, Kampala, Uganda), and incubated at 37 °C in ambient air. After 24 h of incubation, growth characteristics and colony morphology were evaluated. Subsequently, for each plate, mannitol fermenting colonies were selected for further identification. Among the morphologically similar mannitol fermenting colonies, only one characteristic colony was selected for further processing while morphologically distinct colonies were treated uniquely. Additionally, the slow (delayed)-mannitol fermenting colonies suspected to be *S. aureus* were subjected to overnight incubation before discarding.

### Deoxyribonuclease (DNase) and tube coagulase test

Suspected colonies of *S. aureus* were inoculated onto DNase agar (Difco Laboratories, Detroit, Mich.), and incubated at 37 °C for 24 h. The colonies were then flooded with a 1 N HCl solution with the excess acid tipped off. The plates were then read for a clear zone around the colonies within 5 min. A DNase positive result was identified by a strong clear zone around the bacterial streak as previously described [23].

For the tube coagulase test, colonies of test isolates were re-suspended in 2 ml of human plasma in sterile glass test-tubes. The tubes were incubated at 35 °C for 4 h and observed for clot formation and then for an additional 18 h at room temperature if clotting did not occur and then read without agitation. The formation of a firm clot on the tube coagulase test was indicative of *S. aureus* as previously described [24]. Suspect *S. aureus* isolates that were positive on the DNase and tube coagulase test were confirmed using the API staph Ident. system (bioMérieux SA, Marcy l'Etoile, France) following the manufacturer’s instructions as previously described [25].

### Antimicrobial susceptibility testing

The susceptibility testing was performed using the Kirby-Bauer disc diffusion method and interpreted according to the Clinical Laboratory Standard Institute [26] guidelines. The antibiotic discs included penicillin G (10U), oxacillin (1 µg), cefoxitin (30 µg), erythromycin (15 µg), clindamycin (2 µg), gentamicin (10 µg), 12 trimethoprim/sulphamethoxazole (25 µg), tetracycline (30 µg), chloramphenicol (30 µg), ciprofloxacin (5 µg), fusidic acid (10 µg), rifampicin (30 µg), linezolid (30 µg), and tigecycline (15 µg) (Mast group ltd., Merseyside, UK). Interpretative zone diameter for tigecycline (15 µg), fusidic acid (10 µg) not included in CLSI guidelines, were interpreted using the European Committee on Antibiotic Susceptibility Testing [27] guidelines.

*S. aureus* ATCC 25,923 (methicillin-susceptible) and BAA-976 (methicillin resistant) were used as quality control strains and were included in each experiment. Isolates showing resistance to oxacillin and cefoxitin were further confirmed by PCR targeting the *mecA* gene. Susceptibility testing to vancomycin was performed for MRSA strains using the VITEK2 system (BioMerieux, Marcy l ‘Etoile, France) at the veterinary microbiology laboratory, Makerere University. To identify inducible macrolide-lincosamide-streptogramin B (MLSB) resistance phenotype, the D-test was performed according to the CLSI guidelines. A “D-shaped” inhibition zone confirmed inducible resistance to clindamycin (2 µg) using erythromycin (15 µg) [28]. The ATCC strain BAA-977 was used as a quality control strain for ICR and was included with each experiment.

### Nucleic acid extraction

The stored *S. aureus* isolates were sub-cultured onto Columbia based blood agar BA (2% agar and 5% sheep blood) and incubated in ambient air at 37 °C overnight. DNA was extracted using the heat-lysis protocol [29] with a slight modification. A loopful from an 18–24-h old culture on BA was suspended in 200 µl of the Tris buffer, heated at 95 °C for 15 min and then centrifuged at 10,000 g for 5 min. A volume of 50 µl of the supernatant was kept at -20 °C for molecular characterization.

### Confirmation of resistance to cefoxitin and oxacillin by *mecA* PCR

*S. aureus* isolates that showed resistance to oxacillin were screened for the *mecA* gene using end-point PCR as described previously [30]. The PCR reactions contained a final concentration of 1X Super-Therm buffer, 1.5 mM MgCl2, 1.5U Super-Therm Taq polymerase (JMR holdings, London, UK), and 0.2 mM deoxynucleotide 13 triphosphate mix (dNTPs) (Thermo Scientific, Wilmington, USA), and a primer concentration of 0.25 µM for each primer (Table 2).

**Table 2:** Primers used to amplify the mecA gene

Primers 5’ – 3’	5’ – 3’ primer sequence	Amplicon size
*mecA* forward primer	TCCAGATTACAACTTCACCAGG	162 bp
*mecA* reverse primer	CCACTTCATATCTTGTAACG	

A volume of 5 µl DNA template was added to the mixture to make a total volume of 50 µl. The PCR amplification was performed in the Applied Biosystems 2720 Thermal cycler (Applied Biosystems, Carlsbad, USA). The thermal cycling temperatures were as follows: denaturation at 94 °C for 4 min, followed by 30 amplification cycles of 94 °C, 53 °C and 72 °C for 30 s each and final elongation at 72 °C for 3 min. The *S. aureus* ATCC 25,923 control strain and PCR grade water were utilized as the positive and negative controls respectively. The PCR products were loaded on 2% agarose gel in 1% Tris–acetic acid-EDTA buffer at 80 V for 1.5 h and visualized with ethidium bromide.

### Characterization of SCCmec elements by a multiplex end point PCR

The *mec*A-positive *S. aureus* isolates were further characterized based on the SCCmec-element using the multiplex PCR as described previously [31]. The multiplex PCR had a final volume of 50 µl and contained a concentration of 1X Super-Therm buffer, 1.5 mM MgCl2, 1.5U Super-Therm Taq polymerase (JMR holdings, London, UK), 0.2 mM dNTPs (ThermoScientific, Wilmington, USA), and primers as well as control strains for SCCmec-types I-VI. The *mec*A gene was amplified as an internal control using the primers *mecA*P4 and *mecA*P7 (0.4 µM concentration). The primers CIF-F2, CIF-R2, RIF-F10, RIF- R13 were added at a final concentration of 0.6 µM, while the primers ccrB-F2, ccrB-R2, ccrC-F2, ccrC-R2, dcs-F2, dcs-R1, kdp-F1, kdp-R1, mecIP3, mecIP2, SCCmec III J1F, SCCmec J1R, SCCmec VJ1F, SCCmec V J1R were added to a final concentration 0.8 µM using sequences as previously described [31].

Three microliters that contained 5 ng of DNA of each of the samples was added to the PCR mix. The prototypic MRSA control strains for SCCmec types I-VI were used as positive controls: COL (type I [1B]), BK2464(type II [2A]), ANS46 (type III [3A]), MW2 (type IV[2B]), WIS (Type V[5C]) and HDE288 (type VI[4B]). The PCR amplification was performed in a Applied Biosystems 2720 Thermocycler (Applied Biosystems, Carlsbad, USA) as follows;

denaturation at 94 °C for 5 min, followed by 30 amplification cycles of 94 °C, 53 °C and 72 °C for 30 s each and a final elongation step at 72 °C for 4 min. The PCR products were loaded on a 3% agarose gel and electrophoresed at 80 V for 2 h before UV- Ethidium Bromide visualization.

## Data Availability

All the datasets used and /or analyzed during the current study are presented as figures or tables. The authors confirm that there are no other datasets relating to this study that are not presented in the tables or figures.

## Abbreviations

UWA: Uganda Wildlife Authority
CoVAB: College of Veterinary Medicine Animal Resources and Biosecurity
